# Polymorphisms in Autophagy Genes and Susceptibility to Tuberculosis

**DOI:** 10.1371/journal.pone.0041618

**Published:** 2012-08-06

**Authors:** Mario Songane, Johanneke Kleinnijenhuis, Bachti Alisjahbana, Edhyana Sahiratmadja, Ida Parwati, Marije Oosting, Theo S. Plantinga, Leo A. B. Joosten, Mihai G. Netea, Tom H. M. Ottenhoff, Esther van de Vosse, Reinout van Crevel

**Affiliations:** 1 Department of Medicine, Radboud University Nijmegen Medical Centre, Nijmegen, The Netherlands; 2 Nijmegen Institute for Infection, Inflammation and Immunity (N4i), Radboud University Nijmegen Medical Centre, Nijmegen, The Netherlands; 3 Department of Internal Medicine, Faculty of Medicine, Universitas Padjadjaran, Bandung, Indonesia; 4 Health Research Unit, Faculty of Medicine, Universitas Padjadjaran, Bandung, Indonesia; 5 Department of Biochemistry, Faculty of Medicine, Universitas Padjadjaran, Bandung, Indonesia; 6 Department of Clinical Pathology, Faculty of Medicine, Universitas Padjadjaran, Bandung, Indonesia; 7 Department of Infectious Diseases, Leiden University Medical Center, Leiden, The Netherlands; Centro de Pesquisa Rene Rachou/Fundação Oswaldo Cruz (Fiocruz-Minas), Brazil

## Abstract

Recent data suggest that autophagy is important for intracellular killing of *Mycobacterium tuberculosis,* and polymorphisms in the autophagy gene *IRGM* have been linked with susceptibility to tuberculosis (TB) among African-Americans, and with TB caused by particular *M. tuberculosis* genotypes in Ghana. We compared 22 polymorphisms of 14 autophagy genes between 1022 Indonesian TB patients and 952 matched controls, and between patients infected with different *M. tuberculosis* genotypes, as determined by spoligotyping. The same autophagy polymorphisms were studied in correlation with ex-vivo production of TNF, IL-1β, IL-6, IL-8, IFN-γ and IL-17 in healthy volunteers. No association was found between TB and polymorphisms in the genes *ATG10*, *ATG16L2*, *ATG2B*, *ATG5*, *ATG9B*, *IRGM*, *LAMP1*, *LAMP3*, *P2RX7*, *WIPI1*, *MTOR* and *ATG4C*. Associations were found between polymorphisms in *LAMP1* (p = 0.02) and *MTOR* (p = 0.02) and infection with the successful *M. tuberculosis* Beijing genotype. The polymorphisms examined were not associated with *M. tuberculosis* induced cytokines, except for a polymorphism in *ATG10*, which was linked with IL-8 production (p = 0.04). All associations found lost statistical significance after correction for multiple testing. This first examination of a broad set of polymorphisms in autophagy genes fails to show a clear association with TB, with *M. tuberculosis* Beijing genotype infection or with ex-vivo pro-inflammatory cytokine production.

## Introduction

*Mycobacterium tuberculosis* (*M. tuberculosis*), the main cause of tuberculosis (TB) worldwide, is an intracellular pathogen that primarily infects macrophages [1], [2]. This pathogen resides and multiplies within a host-derived phagosome where it persists through interference with phagosome-lysosome biogenesis [3], [4]. Recent studies suggest that autophagy, a homeostatic process involved in nutrient regeneration and immune responses, is involved in intracellular killing of *M. tuberculosis* [3], [5], [6], and that physiological or pharmacological induction of this process *in vitro* (i.e.: with ATP, IFN-γ, vitamin D3) promotes fusion of phagosomes containing *M. tuberculosis* with lysosomes and subsequent killing of the pathogen in autophagic characteristic double-membrane autolysosomes [1], [3], [7]. In addition, intracellular survival of *M. tuberculosis* was shown to depend on its ability to escape or inhibit autophagy [5], [8], and a study by Kumar *et al.* found that genes that regulate intracellular survival of *M. tuberculosis*, regardless of its genotype, are in the autophagy pathway itself or in pathways that affect autophagy [9].

Susceptibility to TB is partly genetically determined and variations in genes involved in the autophagic pathway may affect the host response to *M. tuberculosis* infection. Indeed, mice deficient in autophagy and autophagy related genes were found to be more susceptible to infection with *M. tuberculosis* [10], [11] and human mononuclear cells with certain polymorphisms in autophagy related genes displayed an impaired ability to control *M. tuberculosis* growth [12], [13], thus suggesting that polymorphisms in autophagy and autophagy related genes may be associated with TB. This appears to be the case as various polymorphisms in one autophagy gene *IRGM*, a downstream effector of IFN-γ, have been associated with increased protection against *M. tuberculosis* infection in African-American [14] and Chinese individuals [15] and infection by particular *M. tuberculosis* genotypes in Ghana [16]. In addition, polymorphisms in a number of genes which affect autophagy, such as *P2RX7*, have also been associated with TB [17], [18]. However, to our knowledge, besides *IRGM* no other gene of the autophagy pathway itself has been examined in TB patients. We have therefore examined a selection of autophagy genes in a large cohort of TB patients and healthy controls in Indonesia. Since susceptibility to TB may depend on the interplay between host and mycobacterial genotype [2], [9], [19], we also grouped patients’ *M. tuberculosis* isolates into W-Beijing genotype strains, which account for one-third of all *M. tuberculosis* infections in Indonesia [20], [21], and non-W-Beijing genotypes. Furthermore, in a Caucasian cohort that was genotyped for the 22 SNPs, we measured cytokine production in peripheral blood mononuclear cells (PBMCs) stimulated with *M. tuberculosis*.

## Materials and Methods

### Subject Recruitment

We previously recruited consecutive TB patients diagnosed in two outpatient clinics and two hospitals in Jakarta and Bandung (Indonesia) from January 2001 to December 2006, for a series of genetic studies examining host susceptibility to TB [19], [22], [23].

Diagnosis of pulmonary TB (PTB) was done according to World Health Organization criteria by clinical presentation and chest radiograph examination, followed by confirmation with microscopic detection of acid-fast bacilli in Ziehl-Neelsen-stained sputum smears and positive culture of *M*. *tuberculosis* on 3% Ogawa medium. For *M. tuberculosis* genotype analysis, mycobacterial DNA was extracted by bringing 2 loops of bacterial mass from an *M. tuberculosis* culture in saline solution and subsequently heating it at 95°C for 5 min. *M. tuberculosis* genotype was determined by using a commercially available Spoligotyping kit (Isogen Bioscience, Maarssen, The Netherlands) as previously described [20]. *M. tuberculosis* Beijing genotype was defined as a spoligo-pattern showing hybridization to at least 3 of the 9 spacers 35–43 and absence of hybridization to spacers 1–34. Spoligotyping was done at the Hasan Sadikin Hospital, Bandung, Indonesia. In addition, for quality control purposes, spoligotyping of 10% of the isolates and of all isolates lacking hybridization were also done at Gelre Hospital, Apeldoorn, The Netherlands.

We excluded from the genetic studies patients with a confirmed diagnosis of extra-pulmonary TB (n = 93), diabetes mellitus (fasting blood glucose >126 mg/dL) (n = 139) and HIV-positive subjects (n = 10). Standard regimen for treatment of TB consisted of isoniazid, rifampin, pyrazinamide, and ethambutol (2HRZE/4H3R3) was administered free of charge to all patients according to the Indonesian National TB program.

During the above mentioned period we also recruited 1000 randomly selected age and gender matched, but genetically unrelated control subjects from the same, mostly poor and densily populated areas where TB is abundant. All control individuals were subjected to the same physical examination, blood tests and chest radiography as the TB patients. A total of 952 control subjects were enrolled in the study after excluding individuals with symptoms or chest X-ray abnormalities suggesting active TB or a history of TB.

A structured questionnaire was used for patients and control subjects to record clinical information, age, gender, self and parental ethnicity, socio-economic status and concurrent medical history.

### Ethics Statement

All individuals recruited signed a written informed consent. The study protocol was reviewed and approved by the local institutional review boards of the medical faculty of university of Indonesia, the Eijkman institute for molecular biology in Jakarta in Indonesia and the Medical Ethical Committee Arnhem-Nijmegen in The Netherlands.

### Genotyping

Using NCBI SNP database we selected SNPs in autophagy genes previously associated with TB (*P2RX7*- rs2393799 [17]), other diseases (*ATG16L1*- rs2241880 [24], [25], *ATG5*- rs2245214 [26] IRGM rs72553867 [27] rs4958847 [28] or with a minor allele frequency of at least 5% (Table 1).

**Table 1:** Polymorphisms in autophagy genes studied.

Gene	Gene ID	SNP	Heterozygosity^a^		Disease associated
			Asians^b^	All populations	
ATG10	83734	rs1864183	19%	49%	N.A.
		rs3734114	38%	25%	N.A.
ATG16L1	55054	rs2241880	47%	48%	Inflammatory bowel disease [24] and Croh 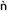 s disease [25]
ATG16L2	89849	rs11235604	N.D.	18%	N.A.
ATG2A	23130	rs77228473	N.D.	N.D.	N.A.
		rs77833427	8%^c^	0.5%	N.A.
ATG2B	55102	rs3759601	29%	42%	N.A.
		rs9323945	42%	16%	N.A.
		rs74719094	N.D.	0.3%	N.A.
ATG5	9474	rs2245214	62%	50%	Systemic lupus erythematosus [26]
ATG9B	285973	rs61733329	17%^c^	4%	N.A.
IRGM	345611	rs72553867	29%^c^	22%	Crohn’s disease [27]
		rs4958847	33%	48	Crohn’s disease [28]
LAMP1	3916	rs9577229	17%^c^	16%	N.A.
LAMP3	27074	rs482912	42%	51%	N.A.
P2RX7	5027	rs2393799	48%^c^	48%	Tuberculosis [17]
WIPI1	55062	rs883541	49%	35%	N.A.
**GWAS**					
MTOR	2475	rs6701524	21%	30%	N.A.
		rs10492975	18%	20%	N.A.
ATG4C	84938	rs10493328	5%	15%	N.A.
		rs10493327	40%	48%	N.A.
		rs10493329	4%	19%	N.A.

a data from dbSNP,

b data from HapMap-HCB (Han Chinese from Beijing),

c data from low coverage pilot panel CHB+JPT (Han Chinese from Beijing and Japanese from Tokyo), N.D.  =  no data available, N.A.  =  no known association.

Blood samples were obtained by venapuncture. Genomic DNA was isolated from EDTA blood of patients, controls and a cohort of healthy volunteers using standard methods, and 5 ng of DNA was used for genotyping. Multiplex assays were designed using Mass ARRAY Designer Software (Sequenom) and genotypes were determined using Sequenom MALDI-TOF MS according to manufacturer’s instructions (Sequenom Inc., San Diego, CA, USA). Briefly, the SNP region was amplified by a locus-specific PCR reaction. After amplification a single base extension from a primer adjacent to the SNP was performed to introduce mass differences between alleles. This was followed by salt removal and product spotting onto a target chip with 384 patches containing matrix. MALDI-TOF MS was then used to detect mass differences and genotypes were assigned real-time using Typer 4 software (Sequenom Inc. San Diego, CA, USA). As quality control, 5% of samples were genotyped in duplicate and each 384-well plate also contained at least 8 positive and 8 negative controls, no inconsistencies were observed. DNA samples of which half or more of the SNPs failed (N = 90) were excluded from analyses. Variants with call-rates below 90% were also excluded from further analyses (n = 0).

For quality control purposes the genotype of at least two samples for each homozygous genotype were confirmed by sequencing using Sanger method with Big Dye Terminator version 3 (Applied Biosystems). After the cycle sequence reaction, the samples were purified by ethanol precipitation and analysed on a 3730 Sequence Analyzer (Applied Biosystems).

**Figure 1:**
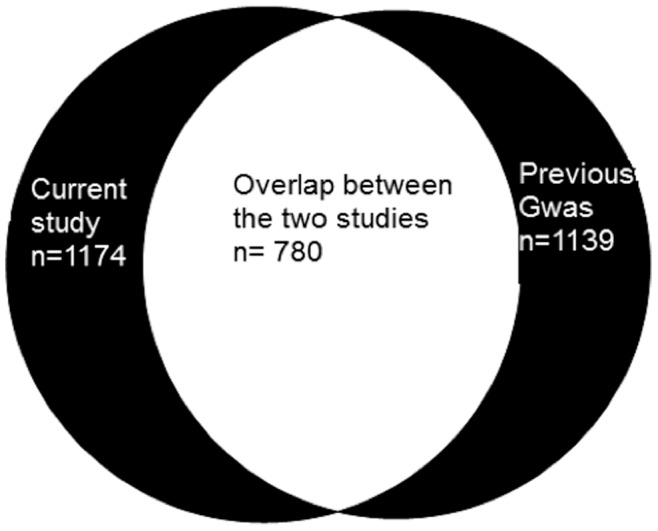
The overlap of study subjects between the current study (n = 1974) and the previous genome-wide association study (GWAS, n = 1139) [**29**].

Previously polymorphisms in various genes were genotyped on a two-stage genome-wide association study (GWAS) using Illumina’s GoldenGate Assay according to manufacturer instructions, aiming to discover genes relevant in pulmonary TB susceptibility in the same Indonesian cohort involved in the current study [29]. Among the SNPs studied, five were in autophagy genes and were included in our data analysis (Table 1). The overlap of study subjects between the current study and the GWAS is shown in Figure 1.

### Cytokine Production by M. tuberculosis Stimulated PBMC

Cells isolated from healthy Caucasian volunteers bearing various genotypes were examined for cytokine production induced by sonicated *M. tuberculosis* H37Rv (n = 67). These individuals were aged 23–73 years, 77% was male and none had a known TB contact. All gave written informed consent, and the study was approved by the Ethical Committee of the Radboud University Nijmegen Medical Centre, Nijmegen, The Netherlands.

Blood samples were obtained by venapuncture. The mononuclear cell fraction was isolated from blood by density centrifugation of blood, diluted 1∶1 in pyrogen-free saline over Ficoll-Paque (Pharmacia Biotech, PA, USA). Cells were washed twice in saline and resuspended in culture medium (RPMI, Invitrogen, CA, USA) supplemented with gentamicin 10 µg/ml, L-glutamine 10 mM, and pyruvate 10 mM. Cells were counted in a Coulter counter (Coulter Electronics) and the number was adjusted to 5×10^6^ cells/ml. A total of 5×10^5^ mononuclear cells in a 100 µl volume of RPMI was added to round-bottom 96-wells plates (Greiner) with or without sonicated *M. tuberculosis* H37Rv (final concentration: 1 µg/ml). After 24 hours, 48 hours (both without serum) or 7 days of incubation (in the presence of 10% serum), supernatants were stored at −20°C. Cytokine concentrations were assessed in the supernatants using enzyme-linked immunosorbent assay (ELISA).

Cytokine measurements of TNF, IL-1β, IL-6, IL-8 (after 24 hours incubation); IFN-γ (after 48 hours incubation), and IL-17 (7 days incubation) were performed in the supernatants using commercial ELISAs from R&D Systems, MN, USA (TNF, IL-1β, IL-8, and IL-17) or Sanquin, Amsterdam, The Netherlands (IL-6 and IFN-γ).

### Statistical Analysis

All data collected from the questionnaires and genotyping were analysed using SPSS version 17.0 (SPSS Inc., Chicago, IL, USA). The Hardy-Weinberg equilibrium (HWE) was checked for each SNP using the program HWE Version 1.10 (Rockefeller University, New York). The program Conting was used to calculate the χ^2^ and the associated values for a contingency table. Patient data were stratified for the *M. tuberculosis* genotype with which they were infected; Beijing or non-Beijing strains and the χ^2^ was calculated with SPSS. Differences in cytokine production were analyzed using the Wilcoxon signed rank test. All statistical analyses were 2-sided, and *P*<0.05 was considered to be statistically significant.

The available number of study subjects allowed us to observe a 5% allele difference between patients and controls for the SNP in IRGM (rs4958847), based on previously reported allele distribution in the general population, a power (β) of 0.80 and a significance level (α) of 0.05.

## Results

### Study Subjects

A total number of 1022 confirmed pulmonary TB patients and 952 age- and gender matched community controls were included in the data analysis. As shown in Table 2, 78% of patients and control subjects were Javanese (a population group with relatively low genetic variance in Indonesia [30]) with similar age, gender distribution, and likelihood of having a BCG scar. Furthermore, both groups also had a similar socioeconomic status (not shown) and previous analysis in this cohort [29] showed that population stratification was minimal.

**Table 2:** Demographic information of the study population.

	TB patients	controls	p value
**Mean age (years)**	33	33	0.7
**Gender male (%)**	53.4	53.2	0.6
**Self reported ethnicity**			0.9
**Javanese**	78.5%	78.3%	
**Mixed (either parent Javanese)**	11.1%	11.8%	
**Non-Javanese**	8.1%	8.2%	
**unknown**	2.3%	1.7%	
**BCG scar present**	44%	49%	0.07

### Association between Polymorphisms in Autophagy Genes and Susceptibility to TB

Polymorphisms rs11235604 (in *ATG16L2*), rs77228473 and rs77833427 (in *ATG2A*), rs74719094 (in *ATG2B*), rs72553867 (in *IRGM*), rs10493328 and rs10493329 (in *ATG4C*) were rare in the study subjects. With the exception of the SNP rs3759601 in *ATG2B* (HWE: 2p  = 0.034), all polymorphisms were in Hardy–Weinberg equilibrium in the healthy controls. The distribution of the alleles for all polymorphisms analyzed in the current study is presented in Table 3. After Chi-square testing we did not detect significant associations between any genetic polymorphism and susceptibility to TB. This was also the case when the largest group (Javanese) was analysed separately (data not shown).

**Table 3:** Distribution of polymorphism allele frequencies in cases and controls.

Gene	SNP	Allele	Frequency in cases (%)	Frequency in controls (%)	P value
	**Autophagy specific genes**				
**ATG10**	rs1864183	A	1450 (86)	1400 (83.3)	0.094
		G	236 (14)	280 (16.7)	
		AA	625 (74.1)	583 (69.4)	
		GA	200 (23.7)	234 (27.9)	
		GG	18 (2.1)	23 (2.7)	
	rs3734114	C	491 (29.2)	502 (29.8)	0.489
		T	1189 (70.8)	1180 (70.2)	
		CC	71 (8.5)	84 (10)	
		TC	349 (41.5)	334 (39.7)	
		TT	420 (50)	423 (50.3)	
**ATG16L1**	rs2241880	C	449 (26.6)	453 (26.8)	0.876
		T	1237 (73.4)	1235 (73.2)	
		CC	62 (7.4)	59 (7)	
		TC	325 (3.6)	335 (39.7)	
		TT	456 (54.1)	450 (53.3)	
**ATG16L2**	rs11235604	CC	835 (99)	839 (99.2)	n.a.
		TC	9 (1)	6 (0.8)	
**ATG2A**	rs77228473	C	1676 (100)	1680 (100)	n.a.
	rs77833427	CC	837 (99.9)	843 (99.9)	n.a.
		TC	1 (0.1)	1 (0.1)	
**ATG2B**	rs9323945	C	1240 (74)	1267 (75.6)	0.566
		T	436 (26)	409 (24.4)	
		CC	464 (55.4)	485 (57.4)	
		TC	312 (37.2)	297 (35.4)	
		TT	62 (7.4)	56 (7.2)	
	rs74719094	TT	842 (99.8)	844 (100)	n.a.
		TG	2 (0.2)		
**ATG5**	rs2245214	C	1017 (59.3)	1006 (60.1)	0.632
		G	697 (40.7)	668 (39.9)	
		CC	294 (35.9)	300 (35,8)	
		GC	395 (46.8)	406 (48.5)	
		GG	145 (17.3)	131 (15.7)	
**ATG9B**	rs61733329	C	1542 (91.4)	1513 (89.4)	0.046
		T	146 (8.6)	179 (10.6)	
		CC	706 (83.6)	676 (79.9)	
		TC/TT	138 (16.4)	170 (20.1)	
**IRGM**	rs72553867	A	83 (4.9)	95 (5.6)	0.389
		C	1605 (95.1)	1742 (94.4)	
		AA/CA	82 (9.7)	93 (11)	
		CC	762 (90.3)	753 (89)	
	rs4958847	A	661 (39.4)	664 (39.6)	0.908
		G	1017 (60.6)	1014 (60.4)	
		AA	131 (15.6)	128 (15.3)	
		GA	399 (47.6)	408 (48.6)	
		GG	309 (36.8)	303 (36.1)	
**LAMP1**	rs9577229	C	1349 (80.9)	1382 (82.3)	0.364
		T	319 (19.1)	298 (17.7)	
		CC	538 (64.5)	567 (65.5)	
		TC	273 (32.7)	248 (29.5)	
		TT	23 (2.8)	25 (3.0)	
**LAMP3**	rs482912	A	762 (45.3)	774 (46.1)	0.89
		G	920 (54.7)	906 (53.9)	
		AA	166 (19.7)	170 (20.2)	
		GA	430 (51.1)	434 (51.7)	
		GG	245 (29.1)	236 (28.1)	
**P2RX7**	rs2393799	C	775 (46)	766 (45.4)	0.932
		T	909 (54)	922 (54.6)	
		CC	181 (21.5)	177 (20.6)	
		TC	413 (49)	412 (49.9)	
		TT	248 (29.5)	255 (29.5)	
**WIPI1**	rs883541	A	655 (38.9)	669 (39.8)	0.866
		G	1027 (61.1)	1011 (60.2)	
		AA	120 (14.7)	125 (14.9)	
		GA	415 (49.4)	419 (49.9)	
		GG	306 (36.0)	296 (35.2)	
	**GWAS**				
**MTOR**	rs6701524	A	1092 (92.5)	988 (91.5)	0.57
		G	88 (7.5)	92 (8.5)	
		AA	505 (85.6)	453 (83.9)	
		AG	82 (13.9)	82 (15.2)	
		GG	3 (0.5)	5 (0.9)	
	rs10492975	A	1059 (89.7)	951 (88.1)	0.25
		G	121 (10.3)	129 (11.9)	
		AA	477 (80.8)	417 (77.2)	
		AG	105 (17.8)	117 (21.7)	
		GG	8 (1.4)	6 (1.1)	
**ATG4C**	rs10493328	A	58 (4.9)	32 (3)	0.09
		G	1122 (95.1)	1048 (97)	
	rs10493327	A	805 (68.2)	765 (70.8)	0.37
		G	375 (31.8)	315 (29.2)	
		AA	273 (46.3)	272 (50.4)	
		AG	259 (43.9)	221 (40.9)	
		GG	58 (9.8)	47 (8.7)	
	rs10493329	A	1124 (95.1)	1034 (96.8)	0.15
		G	58 (4.9)	34 (3.2)	

n.a. not analysed; SNP not polymorphous in this population.

### Association between Polymorphisms in Autophagy Genes and M. tuberculosis Genotype

To examine a possible association between host and mycobacterial genotype, autophagy gene polymorphisms were compared between patients infected with *M. tuberculosis* Beijing genotype and other *M. tuberculosis* genotypes. One hundred and sixty-one patients (33%) were infected with *M. tuberculosis* Beijing genotype strains, 322 with a non-Beijing strain, while no strain information was available for the remainder (n = 540). Patients infected with *M. tuberculosis* Beijing and non-Beijing strains were not significantly different in terms of age, sex, or history of previous tuberculosis treatment. The distribution of the alleles for all polymorphisms among patients infected with *M. tuberculosis* Beijing and non-Beijing strains is shown in Table 4. The polymorphism in *LAMP1* (rs9577229) showed an association with TB caused by *M. tuberculosis* Beijing strains, when the TC was combined with the low prevalent TT genotype (p = 0.02). The same was true for the polymorphism in *MTOR* (rs6701524); when combining the AG with the low prevalent GG genotype, *MTOR* was significantly associated with infection with *M. tuberculosis* Beijing strains (p = 0.02). However, both associations lost statistical significance after correction for multiple testing.

**Table 4:** Polymorphism in autophagy genes according to *M. tuberculosis* genotypes strain.

Gene	SNP	Allele	Frequency in Beijing (%)	Frequency in non-Beijing (%)	P value
	**Autophagy specific genes**				
**ATG10**	rs1864183	A	285 (88)	553 (84)	0.260
		G	39 (12)	105 (16)	
		AA	125 (77.2)	232 (70.5)	
		GA	35 (21.6)	89 (27.1)	
		GG	2 (1.2)	8 (2.4)	
	rs3734114	C	87 (27)	206 (31.2)	0.245
		T	235 (73)	454 (68.8)	
		CC	9 (5.6)	33 (10)	
		TC	69 (42.9)	140 (42.4)	
		TT	83 (51.6)	157 (47.6)	
**ATG16L1**	rs2241880	C	89 (27.5)	163 (24.7)	0.475
		T	235 (72.5)	497 (75.3)	
		CC	14 (8.7)	19 (5.8)	
		TC	61 (37.7)	125 (37.8)	
		TT	87 (53.7)	186 (56.4)	
**ATG16L2**	rs11235604	CC	158 (97.5)	326 (98.8)	0.300
		TC	4 (2.5)	4 (1.2)	
**ATG2B**	rs3759601	C	285 (88.5)	602 (91.5)	0.235
		G	37 (11.5)	56 (8.5)	
		CC	127 (78.9)	274 (83.3)	
		GC/GG	34 (21.1)	55 (16.7)	
	rs9323945	C	273 (72.6)	574 (75.1)	0.661
		T	103 (27.4)	190 (24.9)	
		CC	100 (53.2)	218 (56.8)	
		TC	73 (38.8)	138 (36.3)	
		TT	15 (8)	26 (6.9)	
**ATG5**	rs2245214	C	182 (56.5)	393 (60.5)	0.443
		G	140 (43.5)	257 (39.5)	
		CC	51 (31.7)	122 (37.5)	
		GC	80 (49.7)	149 (45.8)	
		GG	30 (18.6)	54 (16.6)	
**ATG9B**	rs61733329	C	300 (92.9)	597 (90.5)	0.235
		T	24 (7.1)	63 (9.5)	
		CC	139 (85.8)	269 (81.5)	
		TC/TT	23 (14.2)	61 (18.5)	
**IRGM**	rs72553867	CA	19 (11.7)	30 (9.1)	0.359
		CC	143 (88.3)	300(90.9)	
	rs4958847	A	136 (42)	251 (38.3)	0.094
		G	188 (58)	405 (61.7)	
		AA	24 (14.8)	53 (16.2)	
		GA	88 (54.3)	145 (44.2)	
		GG	50 (30.9)	130 (39.6)	
**LAMP1**	rs9577229	C	313 (83.7)	587 (77.2)	0.020
		T	61 (16.3)	173 (22.8)	
		CC	128 (68.4)	226 (59.5)	
		TC/TT	59 (31.6)	154 (40.5)	
**LAMP3**	rs482912	A	149 (46.3)	309 (47)	0.831
		G	163 (50.6)	349 (53)	
		AA	32 (19.9)	72 (21.9)	
		GA	85 (52.8)	165 (50.2)	
		GG	44 (27.3)	92 (28)	
**P2RX7**	rs2393799	C	170 (52.5)	301 (45.9)	0.164
		T	154 (47.5)	355 (54.1)	
		CC	46 (28.4)	74 (22.6)	
		TC	78 (48.1)	153 (46.6)	
		TT	38 (23.5)	101 (30.8)	
**WIPI1**	rs883541	A	124 (38.3)	265 (40.3)	0.547
		G	200 (61.7)	393 (59.7)	
		AA	24 (14.8)	47 (14.3)	
		GA	76 (46.9)	171 (52)	
		GG	62 (38.3)	111 (33.7)	
	**GWAS**				
**MTOR**	rs6701524	A	219 (95.2)	417 (91)	0.023
		G	11 (4.8)	41 (9)	
		AA	105 (91.3)	188 (82.1)	
		AG/GG	10 (8.7)	41 (17.9)	
	rs10492975	A	205 (89.1)	416 (90.6)	0.84
		G	25 (10.9)	44 (9.4)	
		AA	92 (80)	189 (82.5)	
		AG	21 (18.3)	37 (16.2)	
		GG	2 (1.7)	3 (1.3)	
**ATG4C**	rs10493328	A	10 (4.3)	22 (4.8)	0.85
		G	220 (95.7)	436 (91.7)	
	rs10493327	A	158 (68.7)	307 (67)	0.81
		G	72 (31.3)	151 (33)	
		AA	53 (46.1)	103 (45)	
		AG	52 (45.2)	101 (44.1)	
		GG	10 (8.7)	25 (10.9)	
	rs10493329	A	220 (95.7)	432 (95.2)	0.84
		G	10 (4.3)	22 (4.8)	

n.a. =  not analysed; SNP not polymorphous in this population.

### Polymorphisms in Autophagy Genes and M. tuberculosis Induced Cytokine Production

**Figure 2:**
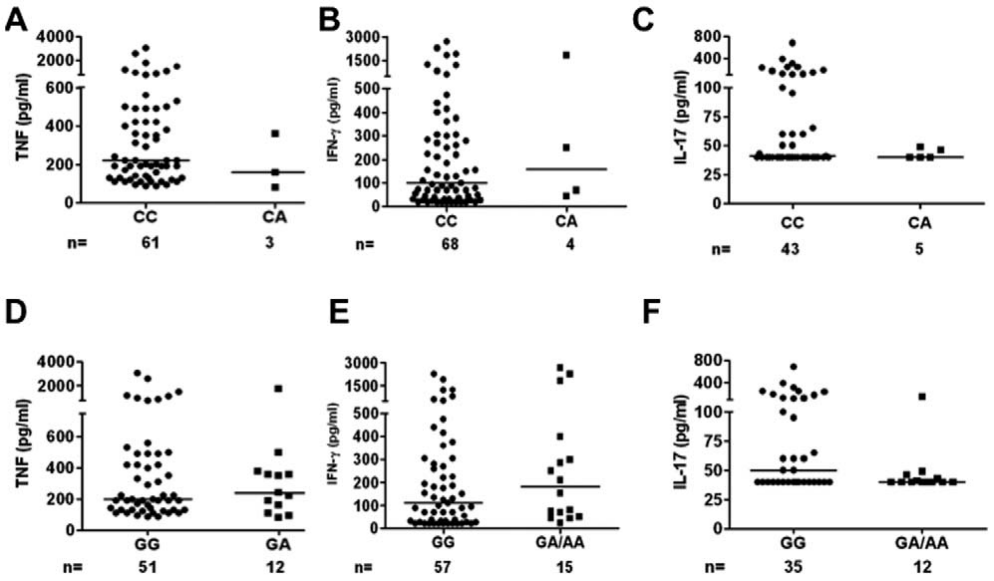
Scatter plots of TNF, IFN-γ, and IL-17 produced after stimulation of PBMC with *M. tuberculosis* stratified for genotypes of both investigated polymorphisms in *IRGM*.

Association between host genotype and *M. tuberculosis* induced cytokine production by PBMC was examined in healthy Caucasian individuals. Table 5 shows the difference in *M. tuberculosis* induced production of TNF, IL-1β, IL-6, IL-8, IFN-γ and IL-17 by PBMC isolated from individuals stratified for different genotype of autophagy related genes. Six of these polymorphisms showed no polymorphic distribution in the Caucasian individuals and could therefore not be analysed. With the exception of *ATG10* (rs1864183), for which a significant difference was found in IL-8 production between individuals with an AA and GG genotype (p = 0.04), no associations were observed between the investigated cytokines and the autophagy related polymorphisms. Figure 2 presents scatter plots of TNF, IFN-γ, and IL-17 stratified for genotypes of both investigated polymorphisms in *IRGM* which was previously linked with susceptibility to TB.

**Table 5:** Variation in cytokine level associated with polymorphisms in autophagy genes.

Gene	SNP	IL-1β^a^	IL-6^a^	IL-8^a^	IL-17^a^	TNF^a^	IFN-γ^a^
**ATG10**	rs1864183	1.4	1.2	1.2^b^	2.1	1.1	1.4
	rs3734114	1.4	1.1	1.1	2.6	1.8	1.2
**ATG16L1**	rs2241880	1.3	1.1	1.1	1.7	1.4	3.3
**ATG16L2**	rs11235604	n.a.	n.a.	n.a.	n.a.	n.a.	n.a.
**ATG2A**	rs77228473	n.a.	n.a.	n.a.	n.a.	n.a.	n.a.
	rs77833427	n.a.	n.a.	n.a.	n.a.	n.a.	n.a.
**ATG2B**	rs9323945	1.1	1.4	1.2	2.5	1.0	3.1
	rs74719094	n.a.	n.a.	n.a.	n.a.	n.a.	n.a.
**ATG5**	rs2245214	1.2	1.3	1.0	1.3	1.4	1.9
**ATG9B**	rs61733329	n.a.	n.a.	n.a.	n.a.	n.a.	n.a.
**IRGM**	rs72553867	1.7	1.0	1.4	2.4	2.2	1.7
	rs4958847	1.3	1.7	1.8	2.9	2.3	2.1
**LAMP1**	rs9577229	n.a.	n.a.	n.a.	n.a.	n.a.	n.a.
**LAMP3**	rs482912	1.2	1.2	1.1	1.6	3.3	3.4
**P2RX7**	rs2393799	1.2	1.1	1.1	1.8	1.3	2.9
**WIPI1**	rs883541	1.4	1.1	1.2	2.9	2.0	4.5

a Values are expressed as the ratio of cytokine production associated with different genotypes (median for all individuals bearing the same genotype), using the genotype groups with the highest and lowest cytokine production.

b Significantly differences (P<0.05) in genotype groups with highest and lowest cytokine production calculated by Wilcoxon signed rank test.

n.a. not analysed; SNP not polymorphous in this population.

## Discussion

*In-vitro* data strongly support a role for autophagy in control of *M. tuberculosis*, and a study involving 2010 patients with pulmonary TB and 2346 control subjects from Ghana has previously reported an association between a polymorphism in the autophagy gene *IRGM* and TB [16]. To further explore a role of autophagy in TB we examined polymorphisms in a number of autophagy genes in TB patients and matched controls from Indonesia. Among almost 2000 subjects, no association was found between TB and 22 SNPs in 14 different autophagy and autophagy related genes, including *IRGM* and *P2RX7* which were previously associated with TB. When TB patients were stratified according to *M. tuberculosis* genotype, associations were observed between SNPs in *LAMP1*, *MTOR* and infection with *M. tuberculosis* Beijing genotype, but statistical significance was lost after correction for multiple testing. No significant correlation was found between *M. tuberculosis* induced cytokine production and genotype of autophagy related genes in a separate cohort of healthy Caucasian volunteers.

IRGM, a downstream effector protein of IFN-γ, induces autophagy and subsequent generation of large autolysosomal organelles as a mechanism for the elimination of intracellular *M. tuberculosis* [31]. In a cohort of 2010 Ghanaian patients and 2346 controls a polymorphism (rs9637876) in *IRGM* was associated with decreased susceptibility to TB caused by *M. tuberculosis* Euro-American (EUAM) lineage, although not for *M. tuberculosis* East-African-Indian (EAI), Beijing, Delhi, *M. africanum* and *M. bovis* lineages [16]. In a study in the US, a polymorphism in *IRGM* (rs10065172) was more common in 370 African-American TB patients compared to controls, but not in 177 Caucasian patients compared to 110 Caucasian controls [14]. We did not find an association between TB, which in Indonesia is mainly caused by the *M. tuberculosis* Beijing lineage, and two different polymorphisms in *IRGM*.

*P2RX7* is an autophagy related gene. It encodes for the P_2_X_7_ receptor, a plasma membrane receptor which mediates ATP-induced autophagy and subsequent intracellular killing of *M. tuberculosis* upon upregulation in mature macrophages [32], [33], [34]. *P2RX7* displays a high genetic heterogeneity [12], and a polymorphism with a C allele at position -762 in the *P2RX7* promoter region was found to have a protective effect against TB in over 300 TB patients and 160 ethnically matched controls subjects from The Gambia [18]. However, no association was found between the same polymorphism and TB in our cohort of Indonesian subjects. It is noteworthy that the protective effect of this polymorphism in Gambian subjects was weak and that it did not correlate with altered receptor expression or activity, suggesting the effect of this SNP might be influenced by other host and pathogen factors [18]. In addition, the relative importance of the role of P_2_X_7_ receptor in the control of *M. tuberculosis* growth is still debated since mice deficient for P_2_X_7_ receptor displayed a similar ability to control pulmonary *M. tuberculosis* infection compared to wild-type mice [35]. Unfortunately, studies on the effect of *P2RX7* polymorphisms in susceptibility to pulmonary TB in humans have not yet been done either *in vivo* or in other ethnic groups.

Polymorphisms in various genes have been associated with TB, but only polymorphisms in *VDR* [36], [37], [38], *NRAMP1* [39], [40] and *MBL* [41], [42] were found to be associated with TB in different geographic regions and ethnic groups. However, the effect of SNPs in these genes varies among racial groups. SNPs in *NRAMP1* were associated with an increased risk of PTB in Gambians [39] but were found to have a protective effect in Cambodians [40], polymorphisms in *MBL* were associated with protection against TB in South Africans [41] but in South Indians increased susceptibility to this disease [42], while SNPs in *VDR* were found to increase susceptibility to PTB in three African countries [36] but to have no effect in Cambodians [40]. As suggested by Fernando et al, these contrasting findings between different ethnic groups may be due to differences in allele frequencies [17]. In addition, the phylogeography of mycobacteria implies that *M. tuberculosis* lineages have become differentially adapted to genetic variations among racial groups [2].

The development of TB is the result of a complex interaction between the host and pathogen influenced by environmental factors [43]. After stratification according to *M. tuberculosis* genotype, we found a suggestive association between TB caused by *M. tuberculosis* Beijing genotype and a polymorphism in *LAMP1,* similar to what we have previously shown for polymorphisms in *SLC11A/NRAMP1* [19]. However, nine major *M. tuberculosis* genotypes have been previously identified in Indonesia [20] and some polymorphisms analysed here may be associated with TB caused by other genotypes not identified in this study.

LAMP1 and LAMP2 are two major protein components of late endosome and lysosome membranes, thought to form a protective barrier against degradation by hydrolytic enzymes [44], [45]. Mice lacking *Lamp2* display impaired autophagy and lysosome biogenesis, while deletion of both *Lamp1* and *Lamp2* is embryonically lethal [44]. However, the contribution of these two lysosomal membrane proteins to phagosomal maturation and killing of intracellular pathogens still needs to be clarified.

Our group recently showed that inhibition of autophagy (genetically or with either siRNA or 3MA) increased IL-1β production [46], [47], [48]. However, with the exception of a SNP in *ATG10* and IL-8, no differences in cytokine production were observed in *M. tuberculosis* stimulated PBMCs of healthy volunteers stratified for genotype of autophagy related genes.

Our paper has several limitations. First and most importantly, no tuberculin skin testing was performed in the control population. However, exposure to tuberculosis must be common in this group, as the majority of controls lived in households of tuberculosis patients, who mostly had a productive cough (98%) for a median of 3 months before first presentation at the TB clinic [49]. Second, as we powered our study on an expected 5% difference in allele frequency between the groups, we cannot exclude possible associations amongst SNPs with a lower frequency.

This is the first paper to investigate the relation of different SNPs in a broad set 14 autophagy genes with susceptibility to TB, as well as with the infecting *M. tuberculosis* genotype and ex-vivo cytokine production. These data further supports the belief that susceptibility to TB has a polygenic nature and polymorphisms in more than one gene may be required to render individuals more or less susceptible to develop active disease.
